# Efficient modification of peroxydisulfate oxidation reactions of nitrogen-containing heterocycles 6-methyluracil and pyridine

**DOI:** 10.3762/bjoc.20.219

**Published:** 2024-10-16

**Authors:** Alfiya Raisovna Gimadieva, Yuliya Zulkifovna Khazimullina, Aigiza Aidarovna Gilimkhanova, Akhat Gaziz:anovich Mustafin

**Affiliations:** 1 Ufa Institute of Chemistry at Ufa Federal Research Center, Russian Academy of Sciences, 71 prosp. Oktyabrya, 450054 Ufa, Russian Federation

**Keywords:** oxidation, 6-methyluracil, peroxydisulfate, phthalocyanine catalysts, pyridine

## Abstract

Nitrogen-containing heterocyclic compounds are widely used in pharmacology due to their pronounced biological activities and low toxicities. The introduction of a hydroxy function into uracil and pyridine molecules has led to compounds with antioxidant, anti-inflammatory, and immunomodulatory activity (3-hydroxy-6-methyl-2-ethylpyridine, 5-hydroxy-6-methyluracil, etc.). One of the successful methods for hydroxylation is peroxydisulfate oxidation. By modifying the Elbs reaction through catalysis and the introduction of additional oxidants, we have been able to significantly increase the yields of practically useful compounds.

## Introduction

The Elbs and Boyland–Sims peroxydisulfate oxidation reactions offer a convenient means of introducing the hydroxy function into phenols and aromatic amines [1]. The oxidation of phenol using peroxydisulfate was first demonstrated by Karl Elbs in 1893 [2], with E. Boyland later expanding this reaction to include aromatic amines [3]. Concurrently, the successful oxidation of several pyrimidine derivatives was also reported [4]. Since then, the reaction has been extensively researched on various classes of compounds such as phenols, coumarins, pyridines, pyrimidines, quinolines, and others. This has resulted in the production of numerous valuable products. The reaction's appeal lies in its simplicity and the fact that there is no requirement to protect sensitive functional groups, thus making the introduction of hydroxy groups into various compound classes an attractive option. However, both reactions suffer from a significant downside – low yields of target products, rendering them unsuitable for industrial application.

Considering the practical value of the products, as well as the simplicity and convenience of the process, our objective is to enhance the efficiency of the Elbs and Boyland–Sims peroxydisulfate oxidation reactions.

## Results and Discussion

**Figure 1:**
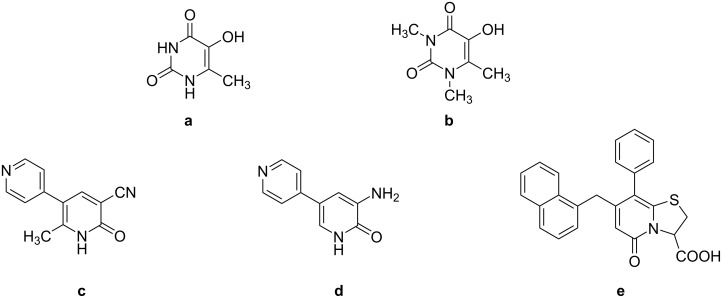
Derivatives of 6-methyluracil and 2-hydroxypyridine demonstrating pharmacological activity: 5-hydroxy-6-methyluracil (**a**), 5-hydroxy-1,3,6-trimethyluracil (**b**), milrinone (**c**), amrinone (**d**), and pilicide (**e**).

We conducted research to enhance the product yield of peroxydisulfate oxidation reactions of specific nitrogen-containing heterocyclic compounds, such as 6-methyluracil (MU), 1,3,6-trimethyluracil (TMU), and pyridine (Py), and the results of the experiments are presented in this article. The hydroxy derivatives of MU and Py, obtained through oxidation followed by acid hydrolysis, possess compelling biological properties, rendering them practically useful. 5-Hydroxy-6-methyluracil (HMU) (Figure 1a) and 5-hydroxy-1,3,6-trimethyluracil (HTMU) (Figure 1b) have been identified as effective antioxidants and radical traps [5–7]. Additionally, 2-hydroxypyridine (HPy) has significant synthetic potential in the design of various bioactive compounds, including drugs and alkaloids [8]. Several drugs are known to contain the 2-pyridone structure, such as the cardiotonic drugs milrinone (Figure 1c) and amrinone (Figure 1d) [9–10], as well as the antibiotic pilicide (Figure 1e) [11–12].

As described in [13] during preliminary experiments, it was determined that room temperature is inadequate for fully oxidizing substrates. The optimal oxidation temperature for uracils **1** and **4** is between 60–65 °C, whereas for pyridines **7** and **9** it is 45 °C. Heating the reaction mixture at a temperature higher than the optimum level causes the substrates to be overoxidized and leads to destruction of the heterocyclic ring. As a result, the yield of the final products decreases. Furthermore, oxidation is incomplete at temperatures lower than the optimal temperature.

**Scheme 1:**
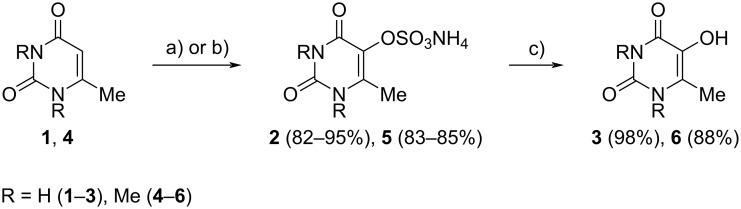
Peroxydisulfate oxidation of 6-methyluracil and 1,3,6-trimethyluracil. Сonditions: a) (NH_4_)_2_S_2_O_8_, 24% NaOH, 60 °C, PcM; b) (NH_4_)_2_S_2_O_8_, 24% NaOH, 60 °C, H_2_O_2_; c) H_2_SO_4_, 80 °C.

The oxidation of MU (**1**), TMU (**4**), and pyridine (**7**) using ammonium peroxydisulfate (APS) was conducted in two different ways: with metallophthalocyanine catalysts present and by including hydrogen peroxide as a co-oxidant (Scheme 1).

Metal–phthalocyanine complexes (PcM) are recognized as catalysts for gentle, particular oxidation reactions under aerobic [14] and H_2_O_2_-based conditions [15–20]. The catalytic activity of metallophthalocyanines originates from their planar, cyclic structure with a developed π-conjugation system. This makes the fifth and sixth coordination sites of the central metal ion available for coordination with the reactant molecules of the catalytic reaction. Additionally, the π-conjugation system facilitates the redistribution of electron density within the reaction complex, thereby lowering the activation barrier of the reaction [21]. The study employed the following oxidation catalysts for phthalocyanines – PcCo, PcFe(II), PcFe(III), PcMn, PcNi, and PcZn.

The use of catalysts reduced the duration of the oxidation reaction and significantly increased the yield of sulfate derivatives.

In the presence of РсМ, the optimal duration for the oxidation reaction of MU (**1**) was found to be 4 hours. When the catalyst was not applied, the yield of MU-5-ammonium sulfate **2** was no more than 15%. Subsequently, upon acid hydrolysis of compound **2**, HMU (**3**) was produced. The addition of PcM to the reaction mixture resulted in a significant increase in the yield of compound **2** [13,22]. The catalysts were added in quantities ranging from 0.00001–0.1 wt %, with the amount of catalyst increasing by a factor of 10 in each successive experiment.

As described in [13] PcFe(II), PcСo, and PcFe(III) exhibited the highest activity in oxidizing reactions of MU (**1**). Addition of these catalysts in the amount of 0.01–0.05 wt % increased the yield of MU-5-ammonium sulfate **2** to 82–95%. The maximum yield of compound **2**, equal to 95%, was obtained when 0.05 wt % PcFe(II) was introduced into the reaction. However, on enhancing the catalyst's quantity to 0.1 wt %, the product yield decreased to 33–45%. Further increase in the quantity of catalyst led to a greater decline in the yield of MU-5-ammonium sulfate, probably due to the destruction of the pyrimidine ring. PcZn turned out to be the least active catalyst, resulting in a yield of MU-5-ammonium sulfate **2** of 72% (0.05 wt %). The catalysts’ activity decreased in the order of PcFe(II) > PcCo > PcFe(III) > PcMn > PcNi > PcZn.

The catalytic oxidation of TMU (**4**, Table 1) displayed a consistent pattern. When 0.01–0.05 wt % of PcСo and PcFe(II) were added to the reaction, a maximum yield of 83–85% of TMU-5-ammonium sulfate **5** was obtained. The catalysts exhibited varying degrees of activity in the following order: PcSo > PcFe(II) > PcFe(III) > PcMn > PcNi > PcZn.

**Table 1:** Yield dependence of 1,3,6-trimethyluracil-5-ammonium sulfate (**5**) on the amount and type of catalyst.^a^

Amount of catalyst, wt %	Yield of **5**, %					
	PcСо	PcFe(II)	PcFe(III)	PcMn	PcNi	PcZn

0	19					
0.001	60	66	60	60	60	48
0.005	67	72	68	66	67	56
0.01	85	83	80	71	65	59
0.02	85	83	81	72	66	61
0.05	85	85	81	72	67	63
0.1	56	55	47	40	39	35
0.2	42	37	35	24	28	27

^a^Mole ratio TMU/NaOH/APS 1:4:1.5; 55 °C; 8 h.

In previous studies, various pyridine derivatives such as 2-pyridone, its derivatives, and 4-pyridone [23–28], were subjected to the Elbs peroxydisulfate oxidation, resulting in yields of up to 38% for the corresponding 5-hydroxy derivatives (2,5-dihydroxy derivatives). Our study marks the first time pyridine has been involved in the peroxydisulfate oxidation reaction.

**Scheme 2:**
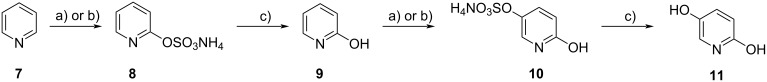
Peroxydisulfate oxidation of pyridine and 2-hydroxypyridine. Сonditions: a) (NH_4_)_2_S_2_O_8_, 24% NaOH, 45 °C, PcM; b) (NH_4_)_2_S_2_O_8_, 24% NaOH, 45 °C, H_2_O_2_; c) HCl, 85–95 °C.

The oxidation of pyridine (**7**) using APS resulted in a single product – pyridin-2-yl ammonium sulfate (**8**). Upon acid hydrolysis, this product yielded HPy (**9**, Scheme 2). The physicochemical and spectral properties of HPy (**9**) are in agreement with those reported in the literature [29].

The most effective catalyst for the pyridine oxidation was found to be РсСо. Its addition to the reaction in the amount of 0.1–0.3 wt % increased the yield of pyridin-2-yl ammonium sulfate (**8**) up to 78–81% (Table 2) [30].

**Table 2:** Yield dependence of pyridine-2-ammonium sulfate (**8**)^a^ and 2,5-dihydroxypyridine (**11**)^b^ on the amount and type of catalyst.

Amount of catalyst, wt %	Yield of **8**/**11**, %					
	PcСо	PcFe(II)	PcFe(III)	PcMn	PcNi	PcZn

0	19/19					
0.01	55/43	36/39	37/37	23/30	26/38	21/23
0.05	67/60	45/54	48/51	45/45	38/47	33/35
0.1	78/71	55/62	57/60	45/49	49/53	42/47
0.15	–/72	–/64	–/62	–/52	–/55	–/49
0.2	81/71	57/63	60/60	50/50	51/53	47/49
0.3	81/–	57/–	58/–	48/–	52/–	46/–

^a^Mole ratio Py/NaOH/APS 1:4:1.5, 45 °C; 10 h; ^b^mole ratio HPy/NaOH/APS 1:4:2; 45 °C; 8 h.

It is notable that even when a large excess of APS was used, only the product of pyridine monohydroxylation, HPy (**9**), was isolated from the reaction mass, probably due to the deactivating effect of the OSO_3_^−^ group on the aromatic ring. Furthermore, 2,5-dihydroxypyridine (**11**) – the pyridine dihydroxylation product – was only obtained after the oxidation of the previously synthesized HPy (**9**, Scheme 2)*.* The overall yield of the 2,5-dihydroxy derivative **11** with PcCo, PcFe(II), and PcFe(III) ranged from 37–72%, with the highest yield (72%) obtained at 0.15 wt % PcCo. Increasing the catalyst quantity did not increase the yield of pyridine **11** further (Table 2).

Despite the numerous works in the field of peroxydisulfate oxidation, there is still no unified view of the reaction mechanism. Consequently, in [31], the assumption of an electrophilic substitution mechanism for the Elbs and Boyland–Sims reactions was made. It has been suggested that a nucleophilic substitution of the peroxide oxygen atom occurs in peroxydisulfate [32]. Regarding phenols (Elbs reaction), there is also a nucleophilic substitution of the phenolate ion. For aromatic amines (Boyland–Sims reaction), a neutral nitrogen atom of the amino group is involved in the formation of an intermediate hydroxylamine derivative.

**Scheme 3:**
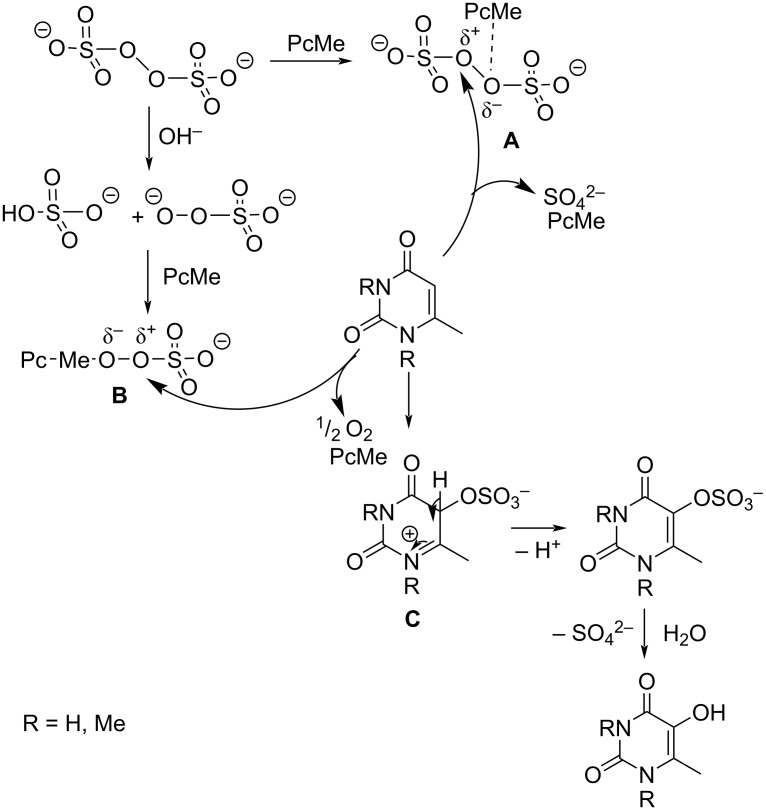
Potential mechanism of peroxydisulfate oxidation of 6-methyluracil and 1,3,6-trimethyluracil.

Scheme 3 demonstrates a possible reaction mechanism using the example of the peroxydisulfate oxidation of MU and TMU catalyzed by PcM. It is proposed that PcM provides the necessary polarization of peroxydi(mono)sulfate ions, thus enabling activated **A** or **B** particles to attack the substrate molecule, resulting in the intermediate σ-complex **C**. A like particle **B** [Pc–Me–O^δ−^–O^δ+^–SO_3_^−^] has been previously described in [33]. The formation of particle **B** is possible via the interaction of the catalyst PcM with the SO_5_^2−^ ion, which is formed by the non-radical decomposition of the peroxydisulfate ion in a strongly alkaline medium [34]:




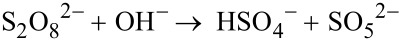




The Elbs and Boyland–Sims reactions were also effectively modified by the use of H_2_O_2_ as a co-oxidant (binary oxidation mixture APS/H_2_O_2_). Adding 2.0–2.3 equiv of H_2_O_2_ (Table 3) [35] resulted in the highest yield of MU-5-ammonium sulfate **2**. In this case, the most effective duration for oxidizing MU were 8 hours, with incomplete conversion observed at shorter reaction times and a slight decrease in the yield of MU-5-ammonium sulfate at durations longer than 8 hours, potentially due to pyrimidine ring destruction. The ideal oxidation of TMU (**5**) was achieved with the addition of 3.0–4.0 equiv H_2_O_2_ (Table 3).

**Table 3:** Yield dependence of 6-methyluracil-5-ammonium sulfate (**2**)^a^ and 1,3,6-trimethyluracil-5-ammonium sulfate (**5**)^b^ on the amount of H_2_O_2_.

Ratio MU/H_2_O_2_, mol	Yield of **2**, %	Ratio TMU/H_2_O_2_, mol	Yield of **5**, %

1:0.85	60	1:1	34
1:1.7	75	1:1.5	35
1:1.91	78	1:2	56
1:2.1	88	1:3	71
1:2.31	85	1:4	71

^a^Mole ratio MU/NaOH/APS 1:4:1.5; 60 °C; 8 h; ^b^mole ratio TMU/NaOH/APS 1:4:1.5; 55 °C; 8 h.

During the oxidation of pyridine (**7**) by the binary oxidation mixture APS/H_2_O_2_ at 45 °C, the yield of the reaction product – Py-sulfate **8** – increased gradually, reaching a maximum of 85% after 10 h of heating [36]. It is essential to include 2–3 equiv of H_2_O_2_ to obtain the best result (Table 4).

**Table 4:** Yield dependence of pyridine-2-ammonium sulfate (**8**) and 2-hydroxypyridine (**9**) on the amount of H_2_O_2_.^a^

Ratio pyridine/H_2_O_2_, mol	Yield, %	

	**8**	**9**

1:0	19	17
1:0,5	50	45
1:1	68	61
1:2	85	77
1:3	85	77

^a^Mole ratio Py/NaOH/APS 1:4:1.5; 45 °С; 10 h.

The analysis of the results obtained from Table 3 and Table 4 indicates that the binary oxidation system of APS/H_2_O_2_ consumes APS more slowly and therefore the substrates (MU, TMU, and Py) are oxidized more completely due to a more efficient consumption of the oxidant (APS). Previously, the oxidation of orotic acid was studied and it was found that the presence of oxygen in the reaction medium affects the yield of the sulfate derivative [37]. Under anaerobic conditions, a low yield of the product is observed with a high rate of APS disappearance, possibly due to side reactions [38]. To create the necessary aerobic conditions, we hypothesize that the inclusion of H_2_O_2_ in the binary oxidation mixture is favorable. This phenomenon occurs as a consequence of the reaction between H_2_O_2_ and the peroxydisulfate ion, leading to the production of oxygen [39]:




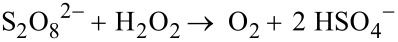




Oxygen accumulation is additionally achieved by hydrogen peroxide self-decay, which is known to be increased in alkaline conditions [40]:




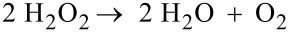




Additionally, it has been reported [41] that hydroxyl radicals, produced from the decomposition of H_2_O_2_, can generate sulfate anion radicals (SO_4_^•−^) [42] during their interaction with sulfate anions. This reaction occurs at a significant rate (*k* = 3.1∙10^8^ min^−1^) [43] and leads to the recombination of peroxydisulfate in the reaction mixture.




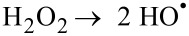







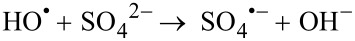







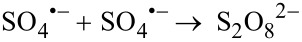




It has been demonstrated that the efficiency of peroxydisulfate oxidation of nitrogen-containing heterocyclic compounds can be enhanced by the introduction of phthalocyanine catalysts or hydrogen peroxide into the reaction mixture. This approach has the potential to significantly increase the yield of the target compounds.

## Conclusion

In conclusion, it is noted that we have identified effective modifications of peroxydisulfate-mediated oxidation reactions that allow us to obtain hydroxylated nitrogen-containing heterocycles in high yields. Our laboratory is currently investigating the application of these modifications to the oxidation of compounds of different classes.

## Experimental

^1^H and ^13^C NMR spectra were recorded on a Bruker Avance III 500 MHz spectrometer at 500.13 MHz (^1^H) and 125.73 MHz (^13^C) with 5 mm QNP sensors at a constant sample temperature of 298 K. The solvents were DMSO-*d*_6_, D_2_O, CDCl_3_ and the internal standard was SiMe_4_. Chemical shifts in the ^13^C and ^1^H NMR spectra are given in parts per million (ppm). Elemental analyses were performed on a CHNS Euro-EA 3000 automatic analyzer. Melting points were determined on combinated Boetius tables. IR spectra were obtained on an IR Prestige-21 Shimadzu spectrophotometer in KBr pellets.

Freshly distilled water, 6-methyluracil (99%, Chemical Line LLC, Russia), pyridine (analytically pure grade, Reakhim LLC, Russia), (NH_4_)_2_S_2_O_8_ (analytically pure grade, Panreac), NaOH (analytically pure grade, Reakhim LLC, Russia), H_2_SO_4_ (reagent grade, LLC Sigma Tech, Russia), dimethyl sulfate (99%, Chemical Line LLC, Russia), chloroform (reagent grade, JSC Khimreaktivsnab, Russia) and ethyl alcohol (reagent grade, LLC TD Khimmed, Russia) were used in this work. The phthalocyanine catalysts (Co, FeII, FeIII, Mn, Ni, and Zn phthalocyanines) were synthesized according to the known procedure [44] from phthalonitrile (pure grade, Merck) and crystalline hydrates of the corresponding metal salts: CoCl_2_·6H_2_O (pure grade, Reakhim LLC, Russia), Ni(NO_3_)_2_·6H_2_O (analytically pure grade, Reakhim LLC, Russia), Fe(NO_3_)_3_·9H_2_O (pure grade, Reakhim LLC, Russia), FeCl_2_·4H_2_O (analytically pure grade, Panreac), MnSO_4_·5H_2_O (analytically pure grade, Reakhim LLC, Russia), and ZnSO_4_·7H_2_O (analytically pure grade, LLC Reachim, Russia); 1,3,6-trimethyluracil was synthesized according to the method [45] from 6-methyluracil, dimethyl sulfate, and NaOH.

### General procedure for the peroxydisulfate oxidation of uracils **1** and **4**

**a) Catalysis by РсМ.** As described in [13] to a three-necked flask of 100 mL capacity, equipped with a mechanical stirrer, a thermometer, and a reflux condenser, containing 10 mL of distilled water, was added 0.023 mol of the corresponding uracil **1** or **4**, followed by the addition of 11 mL of a previously prepared 24% NaOH solution to the obtained suspension. To the obtained thick mass 0.034 mol of (NH_4_)_2_S_2_O_8_ was added in portions under stirring, after which the calculated amount of the corresponding PcM was added. The reaction mixture was stirred at 60 °C for 4 h (for uracil 4–8 h). After cooling the reaction mixture to room temperature, concentrated H_2_SO_4_ was slowly added until pH 6–7 according to litmus paper and left standing for 12 h. The precipitated white crystals were filtered off, washed with water, and dried in air.

Compound **2** yields are given in [13] and compound **5** yields are given in Table 1.

**b) With addition of H_2_O_2_.** To a three-necked flask equipped with a mechanical stirrer, a thermometer, and a reflux condenser containing 10 mL of distilled water was added 0.023 mol of powdered uracil **1** or **4**, followed by the addition of 11 mL of a previously prepared 24% NaOH solution to the obtained suspension. To the obtained thick mass 0.034 mol of (NH_4_)_2_S_2_O_8_ was added in portions under stirring. After complete addition of (NH_4_)_2_S_2_O_8_, 30% H_2_O_2_ (0.048 mol for uracil **1**, 0.069 mol for uracil **4**) was added. The reaction mixture was stirred at 60 °C for 8 h. After cooling the reaction mixture to room temperature, concentrated H_2_SO_4_ was slowly added until pH 6–7 according to litmus paper and left standing overnight (12 h). The precipitated crystals were filtered off, washed with water, acetone, and dried in air. The crude product was recrystallized from water with activated carbon. The yield of compound **2** was 88% and for compound **5** 70%.

### Preparation of compounds

**6-Methyluracil-5-ammonium sulfate (2).** The spectral characteristics are given in [13].

**1,3,6-Trimethyluracil-5-ammonium sulfate (5)**. The spectral characteristics are given in [13].

**General procedure for the hydrolysis of uracils 2 and 5.** As described in [13] in a three-necked flask of 100 mL capacity, equipped with a mechanical stirrer, a reflux condenser, and a dropping funnel, 0.022 mol of compound **2** or **5** was placed, 30 mL of distilled water were added and the mixture heated to 80 °C under constant stirring. Then, 0.022 mol of concentrated H_2_SO_4_ was added and the reaction mixture was stirred at the same temperature for 1 h and cooled. The precipitated crystals were filtered off, washed with cold distilled water to pH 6–7 (2 × 5 mL), and recrystallized from ethanol.

**5-Hydroxy-6-methyluracil (3).** Yield 98%. The spectral characteristics are given in [13].

**5-Hydroxy-1,3,6-trimethyluracil (6).** Yield 88%. The spectral characteristics are given in [13].

**2-Pyridinyl sulfate (8):** a) In a 150 mL three-necked flask equipped with a reflux condenser and a mechanical stirrer, to a solution of 0.06 mol of pyridine in 20 mL of azeotropic solution of water and acetone (1:1) was slowly added to 20 mL of 24% NaOH solution. The reaction temperature was raised to 45 °C and a solution of 0.09 mol of (NH_4_)_2_S_2_O_8_ in 30 mL of water was added. After complete addition of (NH_4_)_2_S_2_O_8_, 0.01 wt % of the catalyst, PcCo, was added. The reaction mixture was stirred at 45 °C for 10 h, cooled to room temperature, evaporated at reduced pressure to 1/3 volume, extracted with ethyl acetate (2 × 20 mL) to remove unreacted pyridine, followd by butanol (3 × 50 mL). The butanol fractions were combined and evaporated to dryness. The residue was washed with hot ethanol, kept for 12 h at 0 °C, the precipitate was decanted, and the filtrate was evaporated to afford 6.33 g (55%) of 2-pyridinyl sulfate as a thick mass of dark brown color. By the same method 2-pyridinyl sulfate (**8**) was prepared in the presence of other catalysts – PcFe(II), PcFe(III), PcMn, PcNi, PcZn. The yields of compound **8** are given in Table 2.

b) In a 150 mL three-neck flask equipped with a reflux condenser, a mechanical stirrer, a solution of 0.06 mol of pyridine in 20 mL of azeotropic solution of water and acetone (1:1) was slowly added to 20 mL of 24% NaOH solution, the reaction temperature was raised to 45 °C and a solution of 0.09 mol of (NH_4_)_2_S_2_O_8_ in 30 mL of water was added. After complete addition of (NH_4_)_2_S_2_O_8_, 0.012 mol of a 30% H_2_O_2_ solution was added. The reaction mixture was stirred at 45 °C for 10 h, cooled to room temperature, evaporated at reduced pressure to 1/3 volume, extracted with ethyl acetate (2 × 20 mL) to remove unreacted pyridine, followed by butanol (3 × 50 mL). The butanol fractions were combined and evaporated to dryness. The residue was washed with hot ethanol, kept for 12 h at 0 °C, the precipitate was decanted, and the filtrate was evaporated to give 9.78 g (85%) of 2-pyridinyl sulfate as a thick mass of dark brown color.

**Pyridine-2(1*H*)-one (9).** 0.0146 mol of 2-pyridinyl sulfate (**8**) was dissolved in 30 mL of distilled water at 90 °C under stirring and after complete dissolution 0.0146 mol of HCl was added. The reaction mixture was heated for 3 h, monitored by TLC (eluent ethanol/ammonia aqueous 4:1), cooled to room temperature, alkalinized with NaHCO_3_ solution to pH 7–8, extracted with chloroform (3 × 10 mL), and the organic layer was dried with MgSO_4_. After removal of the solvent 1.17 g (95%) of pyridin-2(1*H*)-one (**9**) was obtained as a thick mass of light brown color. After recrystallization from ethanol, 1.11 g (90%) of pyridine **9** were obtained as a light yellow powder. Mp 108–110 °С; ^1^H NMR (CDCl_3_) δ 6.16 (dd, 1H, С^5^*H*), 6.38 (d, 1H, С^3^*H*), 7.38 (d, 1H, С^6^*H*), 7.40 (dd, 1H, С^4^*H*), 11.5 (s, 1H, C^1^-O*H*); ^13^С NMR (CDCl_3_) δ 104.8 (С^4^), 119.7 (С^3^), 135.2 (С^6^), 140.8 (С^4^), 162.3 (С^5^). The spectral data is in accordance with [29].

**2,5-Dihydroxypyridine (11).** 0.9 g (0.01 mol) of 2-hydroxypyridine (**9**) was dissolved in a solution of 1.56 g (0.028 mol) of NaOH in 25 mL of distilled water in a flat-bottomed flask fitted with a magnetic stirrer. The flask was placed in an ice bath for cooling and when the temperature of the reaction mixture reached 5 °C, 2.28 g (0.01 mol) of (NH_4_)_2_S_2_O_8_ in 10 mL of distilled water were added. When the reaction temperature reached 23–25 °C, 0.1 wt % PcCo was added. The reaction was kept at room temperature for 20 h. Then, 1.96 g (0.02 mol) of concentrated H_2_SO_4_ were added to the reaction mixture while stirring and the solution was gently boiled for 2 h. After cooling, 40% NaOH solution was added to the reaction mixture until pH 6 and extracted with chloroform (2 × 20 mL). The aqueous layer was evaporated to dryness and treated with hot ethanol. 0.9 g (50%) to obtain a reddish brown glassy solid. After recrystallization in ethanol, light beige crystals of 2,5-dihydroxypyridine (**11**) were obtained. Mp 107–109 °С; ^1^H NMR (CDCl_3_) δ 6.96 (1H, С^3^*H*), 7.16 (1H, С^6^*H*), 7.60 (1H, С^4^*H*), 9.76 (C^5^-O*H*), 10.78 (C^2^-O*H*); ^13^С NMR (CDCl_3_) δ106.2 (*С^3^*), 118.2 (*С^4^*), 128.4 (*С^6^*), 136.5 (*С^5^*), 141.4 (*С^2^*); Anal. calcd for C_5_H_5_NO_2_: C, 53.98; H, 4.67; N, 12.71; found: C, 54.06; H, 4.51; N, 12.62.

## Supporting Information

File 1 NMR spectra of compounds **2**, **3**, and **6**.

## Data Availability

All data that supports the findings of this study is available in the published article and/or the supporting information to this article.
