# Supplementary material for: Efficient modification of peroxydisulfate oxidation reactions of nitrogen-containing heterocycles 6-methyluracil and pyridine
Source: Beilstein J Org Chem. 2024 Oct 16;20:2599–607. doi: 10.3762/bjoc.20.219 (PMC11496723; doi:10.3762/bjoc.20.219)
Supplement: File 1 — NMR spectra of compounds 2, 3, and 6. [file Beilstein_J_Org_Chem-20-2599-s001.pdf]

BEILSTEIN JOURNAL OF ORGANIC CHEMISTRY

Supporting Information

for

Efficient modification of peroxydisulfate oxidation reactions of nitrogen-containing heterocycles 6-methyluracil and pyridine

Alfiya R. Gimadieva, Yuliya Z. Khazimullina, Aigiza A. Gilimkhanova and Akhat G. Mustafin

Beilstein J. Org. Chem. **2024,** *20,* 2599–2607. doi:10.3762/bjoc.20.219

NMR spectra of compounds 2, 3, and 6

License and Terms: This is a supporting information file under the terms of the Creative Commons Attribution License (https://creativecommons.org/licenses/by/4.0). Please note that the reuse, redistribution and reproduction in particular requires that the author(s) and source are credited and that individual graphics may be subject to special legal provisions.
The license is subject to the *Beilstein Journal of Organic Chemistry* terms and conditions: (https://www.beilstein-journals.org/bjoc/terms)

The spectral characteristics of compounds **2**, **3**, **5**, **6** are given in [1], compound **9 -** in [2].

6-Methyluracil-5-ammonium sulfate (2)

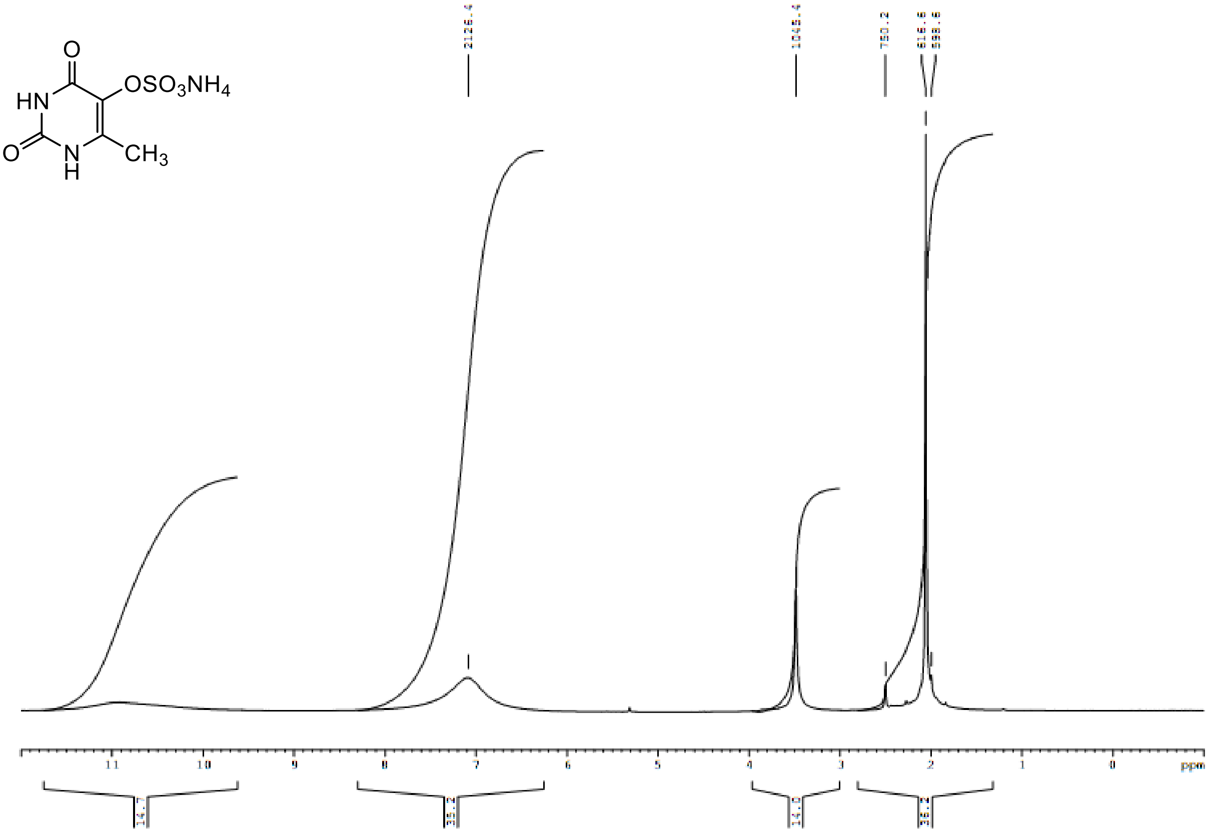

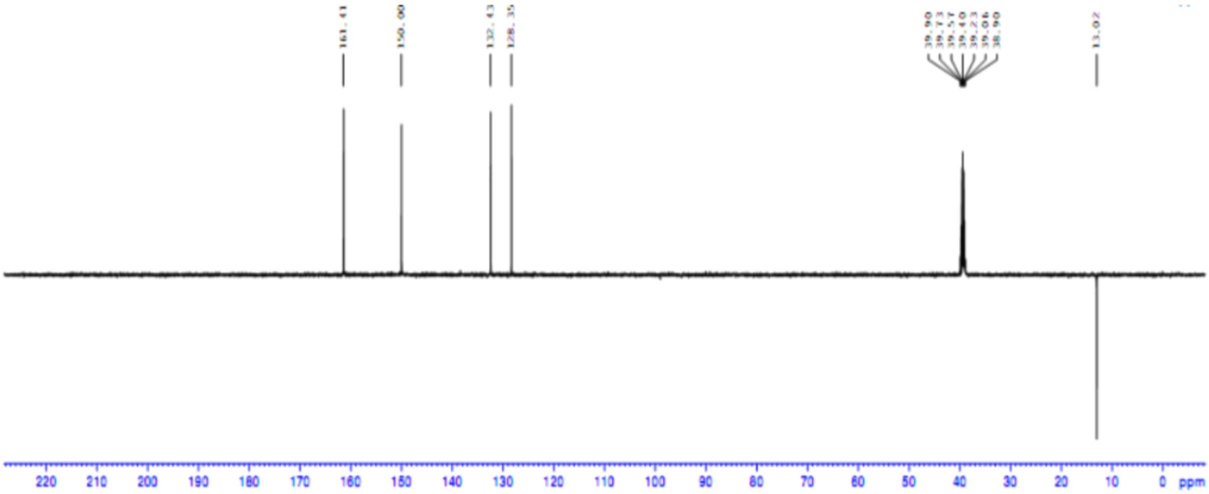

5-Hydroxy-6-methyluracil (3)

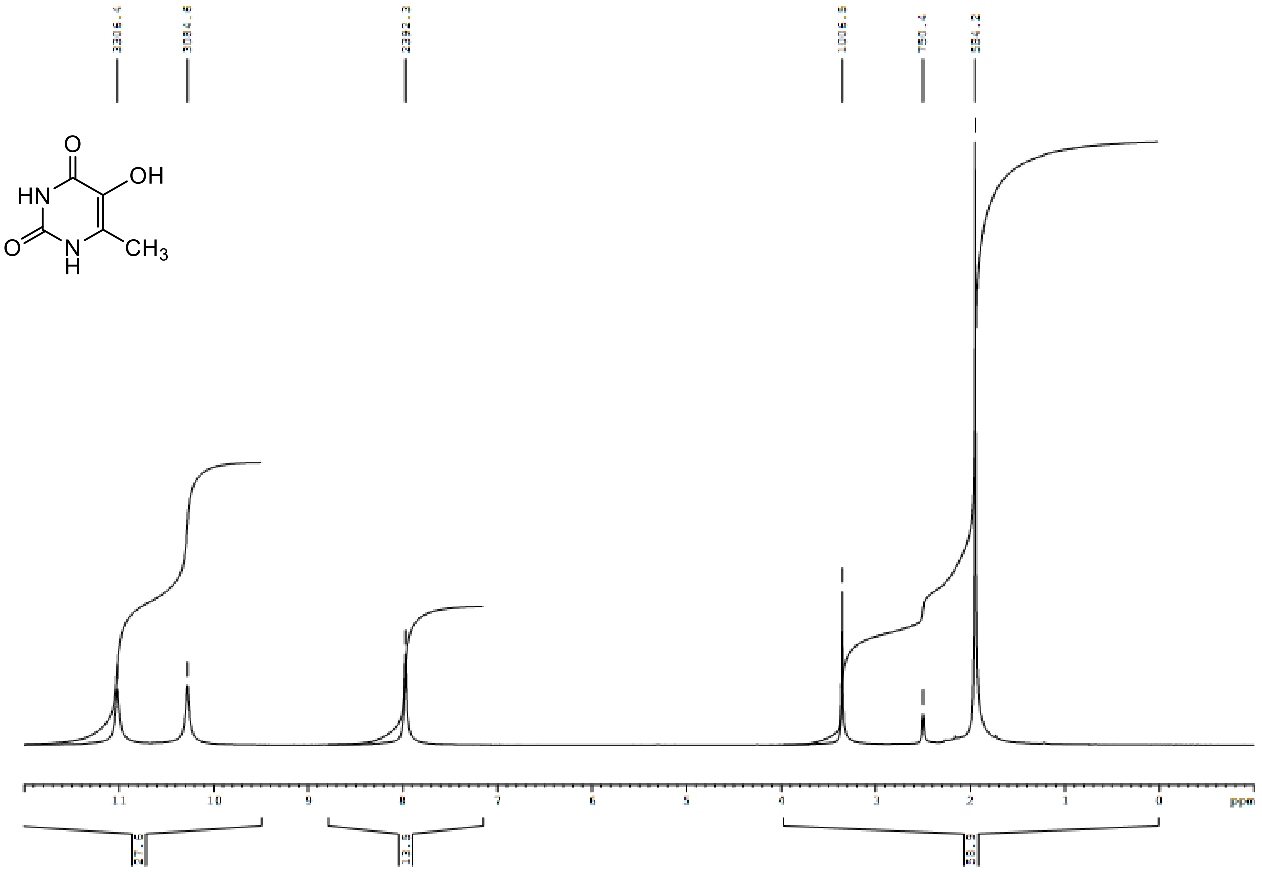

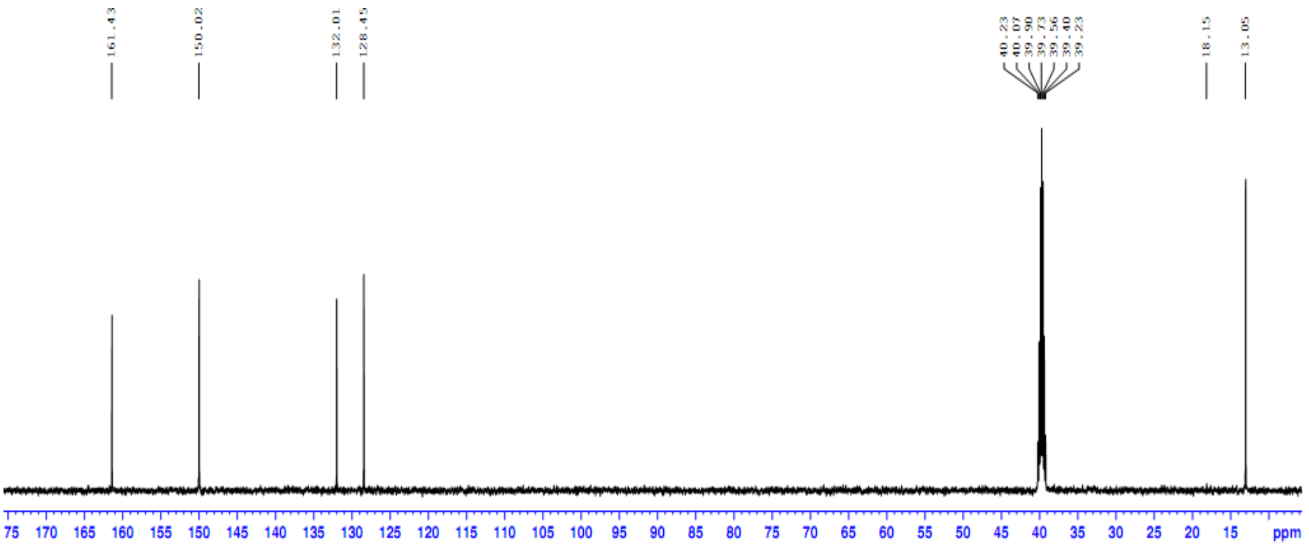

5-Hydroxy-1,3,6-trimethyluracil (6)

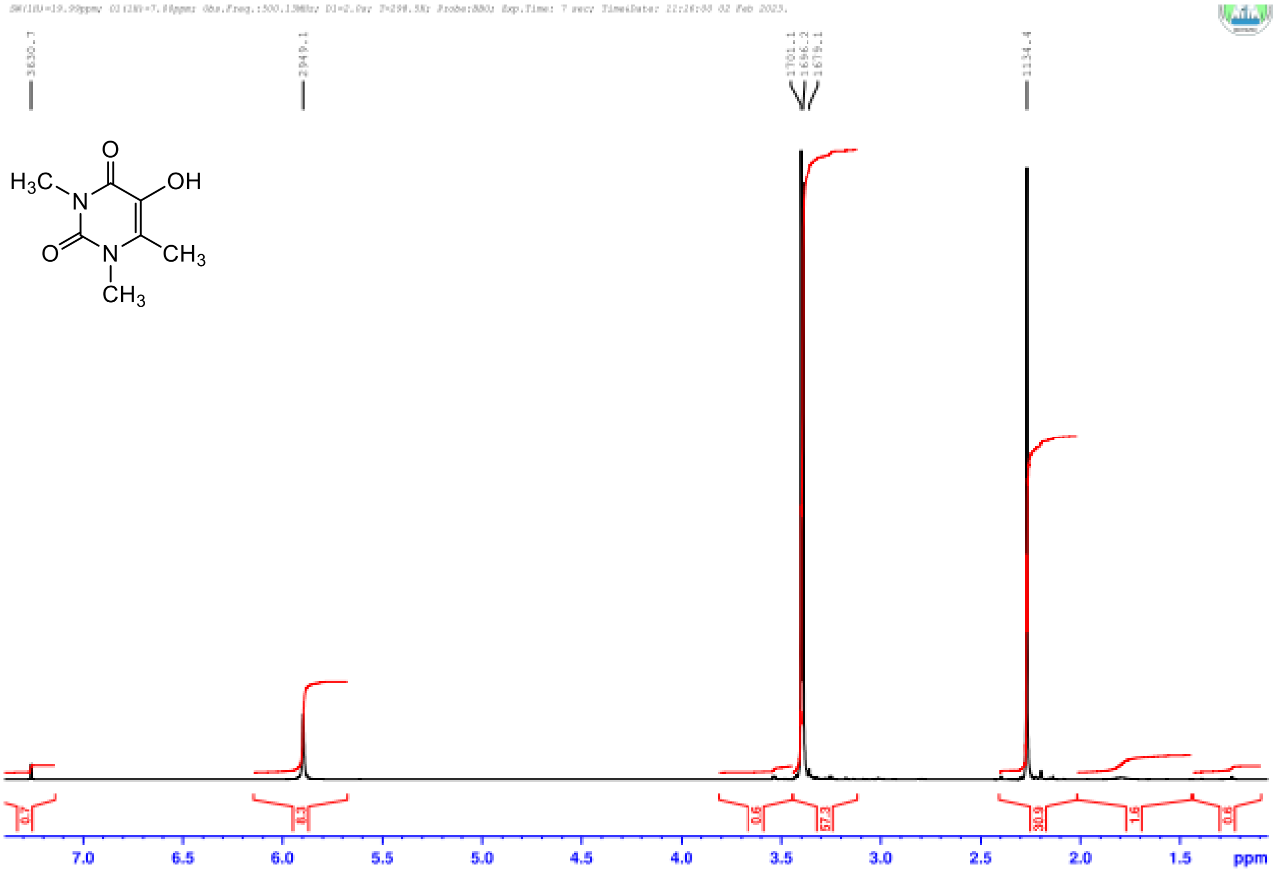

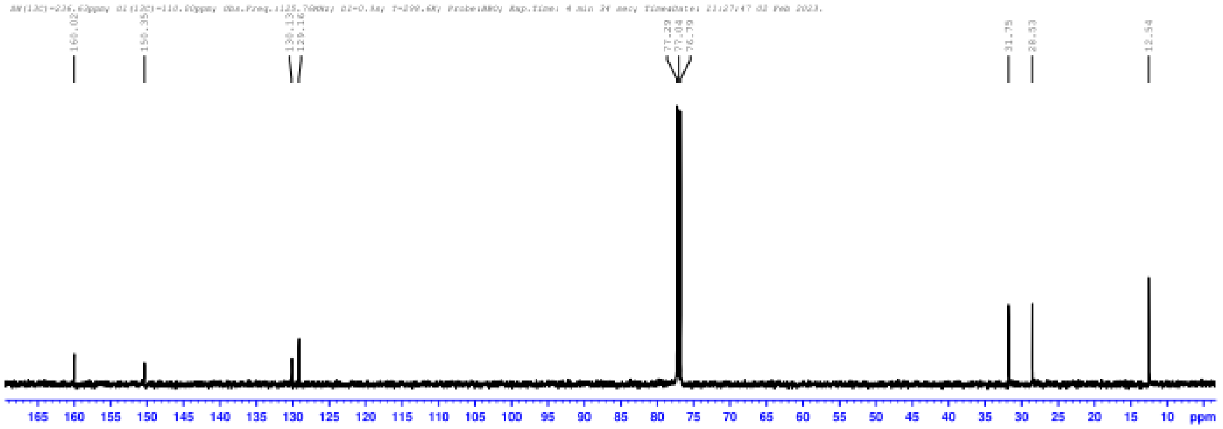

References

1. Gimadieva, A. R.; Khazimullina, Yu. Z.; Abdrakhmanov, I. B;, Mustafin, A. G. *Russ. J. Appl. Chem.*, **2022**, 95, 436-441. DOI: 10.31857/S0044461822030112.

2. Forlani, L.; Cristoni, G.; Boga, C.; Todesco, P.E.; Del Vecchio, E.; Selva, S.; Monari, M. *Arkivoc*, **2002**, **11**, 198-215. DOI: 10.3998/ark.5550190.0003.b18.
